# Reply regarding: Machine learning-based prediction of short- and long-term mortality for shared decision-making in older hip fracture patients

**DOI:** 10.2340/17453674.2026.45733

**Published:** 2026-04-08

**Authors:** Hidde Dijkstra, Cathleen S Parsons, Hanne-Eva van Bremen, Hanna C Willems, Anne A H de Hond, Barbara C van Munster, Job N Doornberg, Jacobien H F Oosterhoff

**Affiliations:** 1 Department of Orthopaedic Surgery, University Medical Centre Groningen, University of Groningen; 2 University Center for Geriatric Medicine, University of Groningen, University Medical Center Groningen, Groningen; 3 Amsterdam Bone Center, Movement Sciences Amsterdam, Amsterdam; 4 Dutch Institute for Clinical Auditing, Leiden; 5 Amsterdam University Medical Centers, location Academic Medical Center, Internal Medicine and Geriatrics, University of Amsterdam, Amsterdam; 6 Amsterdam Public Health Research Institute, Amsterdam; 7 Julius Centre for Health Sciences and Primary Care, University Medical Center, Utrecht; 8 Department of Engineering Systems & Services, Faculty Technology Policy and Management, Delft University of Technology, Delft, the Netherlands

*Sir,* — We appreciate the author’s thoughtful response [1] to our article [2]. As we understand it, their letter highlights that mortality prediction represents only one dimension of what matters in the challenging treatment-related shared decision-making (SDM) in older adults with hip fractures. Clinical decision depends far more on functional outcomes, patient values, and careful communication of prognostic risks. These factors may differ per patient and context.

We fully agree that treatment-related shared decision-making in older adults with hip fractures is complex. Treatment decisions must balance patient-specific factors such as life goals, frailty, and surgical risks. A hip fracture significantly impact a patient’s health-related quality of life and further reduces life expectancy, making prognosis and the decision whether to operate or not particularly challenging in frail, life-limited patients [3]. It would be ideal to have a model in which mortality and functional recovery, such as walking ability or independence, are incorporated. However, until now, we do not have the large numbers to fit an AI tool for both outcomes and more importantly, in clinical decision-making, the chance of mortality can start the talk with the patient about what is important for him or her.

Our Dutch Hip Fracture Audit (DHFA) machine-learning based algorithms were developed to predict 30-day, 90-day, and 1-year mortality in older hip fracture patients, demonstrating adequate model performance. A risk stratification tool that predicts short- and long-term mortality would improve the surgeon’s ability to estimate mortality risk and therefore help determine the most appropriate management of a hip fracture, and may help patients and their families make better-informed decisions. In clinical practice, a higher short-term mortality risk can serve as a meaningful starting point for conversations concerning what truly matters to the patient. Rather than providing an exact or deterministic answer, such predictions function as a support tool to structure the dialogue, clarify values and expectations, and explore treatment preferences. As outlined in our discussion, these predictions should always be embedded within the clinical context and shared decision-making and never be used in isolation.

In our previous research, we found that clinicians consistently emphasize that AI predictions must be contextualized and weighted with clinical judgment and patient specific factors, underscoring the importance of embedding mortality estimates within SDM and the clinical context [4].

To advance trust and clinical adoption of AI based decision support in orthopedic trauma, prospective evaluation of predictive performance and real world decision making impact is essential. In our ongoing follow up study, our purpose is to prospectively evaluate how the DHFA mortality prediction algorithms perform in clinical practice and how their use influences treatment decisions, patient centered outcomes, and human–AI interaction in hip fracture management. We thank the author for emphasizing the importance of contextualizing mortality prediction within SDM. We share this view and aim to further strengthen the clinical relevance of our work by integrating functional and patient reported outcomes in future research.

Sincerely,

**Hidde Dijkstra ^1,2^, Cathleen S Parsons ^2,8^, Hanne-Eva van Bremen ^3-5^, Hanna C Willems ^3,5,6^, Anne A H de Hond ^7^, Barbara C van Munster ^2^, Job N Doornberg ^1^, and Jacobien H F Oosterhoff ^1^**

*^1^ Department of Orthopaedic Surgery, University Medical Centre Groningen, University of Groningen;*

*^2^ University Center for Geriatric Medicine, University of Groningen, University Medical Center Groningen, Groningen;*

*^3^ Amsterdam Bone Center, Movement Sciences Amsterdam, Amsterdam;*

*^4^ Dutch Institute for Clinical Auditing, Leiden;*

*^5^ Amsterdam University Medical Centers, location Academic Medical Center, Internal Medicine and Geriatrics, University of Amsterdam, Amsterdam;*

*^6^ Amsterdam Public Health Research Institute, Amsterdam;*

*^7^ Julius Centre for Health Sciences and Primary Care, University Medical Center, Utrecht;*

*^8^ Department of Engineering Systems & Services, Faculty Technology Policy and Management, Delft University of Technology, Delft, the Netherlands*

*Correspondence: j.h.f.oosterhoff@umcg.nl*

## References

[1] Yıldırım E. Letter to the Editor – Comment on: Machine learning-based prediction of short- and long-term mortality for shared decision-making in older hip fracture patients. 2026; 97: 240. doi: 10.2340/17453674.2026.4536241950115

[2] Dijkstra H, Parsons C S, van Bremen H-E, Willems H C, De Hond A A H, C Van Munster B, et al. Machine learning-based prediction of short- and long-term mortality for shared decision-making in older hip fracture patients: the Dutch Hip Fracture Audit algorithms in 74,396 cases. Acta Orthop 2025; 96: 521-8. doi: 10.2340/17453674.2025.44248 40622227 PMC12231630

[3] Loggers S A I, Willems H C, Van Balen R, Gosens T, Polinder S, Ponsen K J, et al; Group FHS. Evaluation of quality of life after nonoperative or operative management of proximal femoral fractures in frail institutionalized patients: The FRAIL-HIP Study. JAMA Surg 2022; 157(5): 424-44. doi: 10.1001/jamasurg.2022.0089 35234817 PMC8892372

[4] Parsons C S, Zuiderwijk A, Orchard N A, Oosterhoff J H F, de Reuver M. Task-technology fit of artificial intelligence-based clinical decision support systems: a review of qualitative studies. BMC Med Inform Decis Mak 2025; 25(1): 397. doi: 10.1186/s12911-025-03237-8 41152858 PMC12570768

